# Supplementary material for: Rapid turnover of life-cycle-related genes in the brown algae
Source: Genome Biol. 2019 Feb 14;20:35. doi: 10.1186/s13059-019-1630-6 (PMC6374913; doi:10.1186/s13059-019-1630-6)
Supplement: Supplementary file 3 — Protein alignments, corresponding Gblocks html file and CDS sequences for the lineage conserved gametophyte-biased genes. (ZIP 2109 kb) [file 13059_2019_1630_MOESM3_ESM.zip › Con_GA_alignments/Ec-27_004510.aa_ali.fasta-gb.htm]

Ec-27\_004510.aa.fasta.trans.aa\_ali.fasta


## Gblocks 0.91b Results

Processed file: **Ec-27\_004510.aa.fasta.trans.aa\_ali.fasta**  
Number of sequences: **2**  
Alignment assumed to be: **Protein**  
New number of positions: **291** (selected positions are underlined in blue)

```
                         10        20        30        40        50        60
                 =========+=========+=========+=========+=========+=========+
Ec-27_004510     MSRGRSVVISSSSPATYYGGDEHDEEDPLGLLRG----TSADIHDGVGILAGSSSLLRSH
MSTRG.41832.1    MSSGRSTVDVSLAAVSAFH---NDDEDPLGLLPGALRTSHGDVHDRVDERRSSASLYFSM
                 ###########            ###########       ################## 


                         70        80        90       100       110       120
                 =========+=========+=========+=========+=========+=========+
Ec-27_004510     -----TPVRAEVGCE--SGDPREVIDAILRSSKTVRDQVSSRQARTLDSYKAENKLLYKE
MSTRG.41832.1    GPETTTNLRAAGDCYDGSGDPRKAIDAMLRSSKAVRDQVSLRQAQALSTYKAENQQLHKD
                                  ###########################################


                        130       140       150       160       170       180
                 =========+=========+=========+=========+=========+=========+
Ec-27_004510     LSLLPPTYSQYRAVRERVASRRRQRNILETA--GPTASAPICHPLNFHLNG-AEAQVSNE
MSTRG.41832.1    LSLLPPTYSQYKAVRQRASSSRIRRSSPPMSAGLPGAPASVSHPLNFHLHGGAGSDNGMG
                 #########################         #################         


                        190       200       210       220       230       240
                 =========+=========+=========+=========+=========+=========+
Ec-27_004510     DDCSQPECIDFGLTIE-ASELGN-TRALEDEKERGGDNGSMEGKTG-LGLECGEGGDNTT
MSTRG.41832.1    GPNNEGEGPPTGLPAGGATGLEKESRVSEGEQVGAGGNGRRAAAGGGEGLGEGNRGDKSS
                                          #####################  ############


                        250       260       270       280       290       300
                 =========+=========+=========+=========+=========+=========+
Ec-27_004510     SHGRPSLEEGGSVSMDEEPANERPSSGWGRRCSTLARAGSASETPTGRKTAPPPLQRVEA
MSTRG.41832.1    LDGG-LVEEGGGVSGGEEVAKERA---GGKRFSTLARAGSASENTMARTLWPFRL---K-
                 ###    ################     ###########################     


                        310       320       330       340       350       360
                 =========+=========+=========+=========+=========+=========+
Ec-27_004510     RRGEDVPAALWRSKVRGRQMVFSLLEAARIEEEDMEEALLACPAST-----SEGG--GRG
MSTRG.41832.1    PSGEGDIPALRRGKVRGREMVFALLEAARVEEEDMEEMFSASRPRTPMSEDDEEGEEGRR
                   ############################################           ###


                        370       380       390       400       410       420
                 =========+=========+=========+=========+=========+=========+
Ec-27_004510     GREAAFHSLDEIRRRNARELDALMAEYDPRPSTNLPQHTLDRRLRKSLPWPEITDEVVAG
MSTRG.41832.1    RREMAFMRLDEARRRNARELDALMVEYDARPSRCRRGHALN-KLRKALPWPA--------
                 ########################################                    


                        430       440       450       460       470       480
                 =========+=========+=========+=========+=========+=========+
Ec-27_004510     GNRDAASCRHAVLDQRKTVGQGGQGGGGGGGGGGGGGGGGEHAGADIDNGCNGTECAGGD
MSTRG.41832.1    ------------------------------------------------------------
                                                                             


                        490       500       510       520       530       540
                 =========+=========+=========+=========+=========+=========+
Ec-27_004510     REQCHGEGGGEGAWSLSRSGGAGGRAAVDGKAIGGDKGEGGGEIGRSDQGDDDARGGEAR
MSTRG.41832.1    ------------------------------------------------------------
                                                                             


                        550       560       570       580       590       600
                 =========+=========+=========+=========+=========+=========+
Ec-27_004510     GEAVAGAGSGASATAAAGRGTEKLHPESSPCRTSVSPSCGADAADNDAEDTTVAISASPT
MSTRG.41832.1    ------------------------------------------------------------
                                                                             


                        610       620       630       640       650       660
                 =========+=========+=========+=========+=========+=========+
Ec-27_004510     KGAATEDGGPARGGVEGGDETKRLETGQRKKPTLPDPNQVNGSVMADPFNMDHIKDLPGL
MSTRG.41832.1    ------------------------------------------------------------
                                                                             


                        670       680       690       700       710       720
                 =========+=========+=========+=========+=========+=========+
Ec-27_004510     LGGDKRGLSRLVSDTVSYIDPAFPNLEFHRRAAMDRAGYFMPGYKPQVISPGPAAGAYWL
MSTRG.41832.1    ------------------------------------------------------------
                                                                             


                        730       740       750       760       770       780
                 =========+=========+=========+=========+=========+=========+
Ec-27_004510     ERRQSYRVKMSAPPSPGRASTSTSNAGRRKAPQQRPRRRSPQHNNSQHRPNVTASTSTPL
MSTRG.41832.1    ------------------------------------------------------------
                                                                             


                        790       800       810       820       830
                 =========+=========+=========+=========+=========+=======
Ec-27_004510     ADNDRARPSAVLRSKYDGKIAGTTARPIRATRTATASATTRRGPPQGIKGGEKERGK
MSTRG.41832.1    ---------------------------------AAATATT-----------------
```

```
Parameters used
Minimum Number Of Sequences For A Conserved Position: 2
Minimum Number Of Sequences For A Flanking Position: 2
Maximum Number Of Contiguous Nonconserved Positions: 8
Minimum Length Of A Block: 10
Allowed Gap Positions: None
Use Similarity Matrices: Yes
```

```
Flank positions of the 11 selected block(s)
Flanks: [1  11]  [24  34]  [42  59]  [78  145]  [155  171]  [206  226]  [229  243]  [248  263]  [269  295]  [303  346]  [358  400]  

New number of positions in Ec-27_004510.aa.fasta.trans.aa_ali.fasta-gb:  291  (34% of the original 837 positions)
```
